# Effects of continuous use of metformin on cardiovascular outcomes in patients with type 2 diabetes after acute myocardial infarction: A protocol for systematic review and meta-analysis

**DOI:** 10.1097/MD.0000000000025353

**Published:** 2021-04-16

**Authors:** Chuanwen Shen, Shuying Tan, Jun Yang

**Affiliations:** Department of Emergency, Qianjiang Central Hospital of Chongqing, Chongqing, China.

**Keywords:** acute myocardial infarction, meta-analysis, metformin, protocol, type 2 diabetes mellitus

## Abstract

**Background:**

To our knowledge, no meta-analyses or reviews have investigated the efficacy and safety of metformin on cardiovascular outcomes after acute myocardial infarction (AMI) in patients with type 2 diabetes mellitus (T2DM). We thus conduct a high-quality systematic review and meta-analysis to assess the efficacy and safety of metformin on cardiovascular outcomes after AMI in patients with T2DM.

**Methods:**

In this systematic review and meta-analysis, we will search PUBMED, Scopus, EMBASE, and Cochrane Library databases through April, 2021. The study is structured to adhere to PRISMA guidelines (i.e., Preferred Reporting Items for Systematic Reviews and Meta-analyses). The literature search, data extraction, and quality assessments are conducted independently by 2 authors. Outcome measures include all-cause mortality; complications such as acute kidney injury, lactic acidosis, hospitalization for AMI or stroke, or death. Where disagreement in the collection of data occurs, this is resolved through discussion. Review Manager Software (v 5.3; Cochrane Collaboration) is used for the meta-analysis. Two independent reviewers will assess the risk of bias of the included studies at study level.

**Results:**

It is hypothesized that metformin use at the post-AMI is associated with decreased risk of cardiovascular disease and death in patients with T2DM.

**Conclusions:**

This study expects to provide credible and scientific evidence for the efficacy and safety of metformin on cardiovascular outcomes after AMI in patients with T2DM.

**Registration number:**

10.17605/OSF.IO/S3MBP.

## 1 Introduction

Type 2 diabetes mellitus (T2DM) is a very common chronic disease worldwide that is associated with an increased risk of mortality and cardiovascular mortality. The incidences of T2DM, tumor, cardiac, and cerebral vascular diseases in China occupy the top 3 in the noncommunicable diseases.^[1,2]^ Meanwhile, T2DM is cardiovascular “risk equivalents,” with acute myocardial infarction (AMI) being the most common among T2DM complications. Intensive glucose control demonstrates decreased development of microvascular complications and even macrovascular complication with early intervention.^[3]^

Metformin, an oral antidiabetic drug of the biguanide class, exerts its effect by increasing gluconeogenesis and peripheral glucose uptake. A large randomized, multicenter trial showed that metformin users had a lower risk of AMI compared with participants on the diet alone.^[4]^ Moreover, despite achieving similar glycemic control, metformin users had lower all-cause and cardiovascular mortality than sulfonylurea and insulin users. However, the role of metformin in reducing cardiovascular disease remains controversial, and in recent meta-analyses, no cardiovascular results have reached statistical significance.^[6–8]^

Nevertheless, the potential of metformin to confer acute cardioprotective effects in AMI has been well established in the preclinical literature. It has been hypothesized that the favorable effects of pretreatment with metformin in patients with AMI relate to cardioprotection against IRI, independent of its hypoglycaemic actions.^[9,10]^ However, to our knowledge, no meta-analyses or reviews have investigated the efficacy and safety of metformin on cardiovascular outcomes after AMI in patients with T2DM. We thus conduct a high-quality systematic review and meta-analysis to assess the efficacy and safety of metformin on cardiovascular outcomes after AMI in patients with T2DM. It is hypothesized that metformin use at the post-AMI is associated with decreased risk of cardiovascular disease and death in patients with T2DM.

## 2 Materials and methods

### 2.1 Study registration

The systematic review protocol has been registered on Open Science Framework registries. The registration number is 10.17605/OSF.IO/S3MBP. The systematic literature review is structured to adhere to PRISMA guidelines (i.e., preferred reporting items for systematic reviews and meta-analyses), which include requirements deemed essential for the transparent reporting of results. Ethical approval and patient consent are not required because this study is a literature-based study. We will update our protocol for any changes in the entire research process if needed.^[11]^

### 2.2 Data sources and search strategy

**Figure 1:**
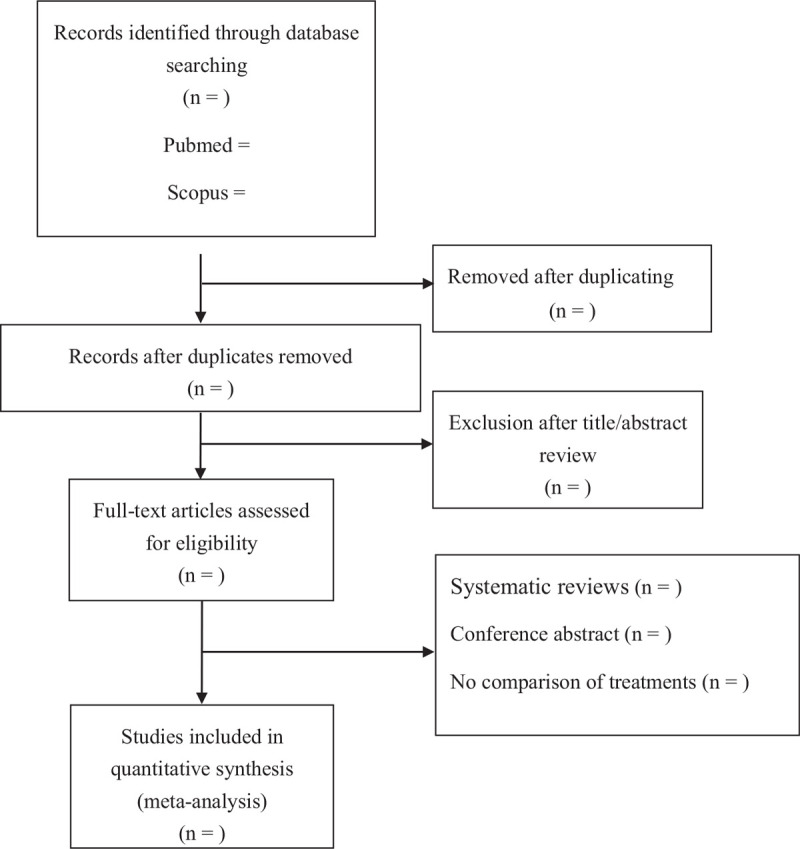
Flow diagram of study identification.

We will search PUBMED, Scopus, EMBASE, and Cochrane Library databases through April, 2021. Search algorithm are identified as follows: (type 2 diabetes) OR (diabetes mellitus) OR (DM) AND (acute myocardial infarction) OR (AMI) AND (metformin). The literature search, data extraction, and quality assessments are conducted independently by 2 authors. We also search references cited in all included articles to avoid missing other relevant articles. If the effective data are not included in the original articles, we will contact the authors to get them. The studies are screened and evaluated by 2 authors independently for eligibility. Flow diagram of study identification is shown in Figure 1.

### 2.3 Eligibility criteria

The study included in our meta-analysis have to meet all of the following inclusion criteria:

1. all clinical trials to assess the efficacy and safety of metformin in the treatment of T2DM after AMI were considered eligible for analysis;
2. adult T2DM patients with a duration of at least 12 weeks (with a glycated hemoglobin level of 7.0% or more);
3. comparing metformin with another antidiabetic therapy or placebo, the cases with metformin treatment and controls with another antidiabetic therapy or placebo;
4. reporting the available data on cardiovascular events.

Studies with overlapping data or insufficient data to calculate or extract effect estimates will be excluded. Case reports, biochemical trials, letters, and reviews will also be eliminated. Articles are exported to EndNote, and duplicates remove. Two independent authors screen the titles and abstracts of potentially relevant studies to determine their eligibility based on the criteria.

### 2.4 Data extraction

Two independent authors extract the following descriptive raw information from the selected studies: study characteristics such as author, publication year, study design; patient demographic details such as patients’ number, average age, body mass index, and gender ratio. Outcome measures include all-cause mortality; complications such as acute kidney injury, lactic acidosis, hospitalization for AMI or stroke, or death. Where disagreement in the collection of data occurs, this is resolved through discussion. If the data are missing or can not be extracted directly, we will contact the corresponding authors to ensure that the information integrated. If necessary, we will abandon the extraction of incomplete data.

### 2.5 Statistical analysis

Review Manager software (v 5.3; Cochrane Collaboration) is used for the meta-analysis. Extracted data are entered into Review Manager by the first independent author and checked by the second independent author. Risk ratio with a 95% confidence interval or standardized mean difference with 95% CI are assessed for dichotomous outcomes or continuous outcomes, respectively. The heterogeneity is assessed by using the *Q* test and I^2^ statistic. An I^2^ value of <25% is chosen to represent low heterogeneity and an I^2^ value of >75% to indicate high heterogeneity. All outcomes are pooled on random-effect model. A *P* value of <.05 is considered to be statistically significant.

### 2.6 Quality assessment

In order to achieve a consistency (at least 80%) of risk of bias assessment, the risk of bias assessors will preassess a sample of eligible studies. Results of the pilot risk of bias will be discussed among review authors and assessors. Two independent reviewers will assess the risk of bias of the included studies at the study level. We will follow the guidance in the latest version of Cochrane Handbook for systematic reviews of interventions when choosing and using tools to assessing risk of bias for randomized trials (version 2 of the Cochrane risk of bias tool for randomized trials, RoB 2) and nonrandomized trials (the Risk Of Bias In nonrandomized Studies of Interventions, ROBINS-I tool). Any disagreements will be discussed and resolved in discussion with a third reviewer. Studies with high risk of bias or unclear bias will be given less weight in our data synthesis.

## 3 Discussion

T2DM is a chronic metabolic disorder that results from defects in both insulin secretion and insulin action. Meanwhile, T2DM is cardiovascular “risk equivalents,” with AMI being the most common among T2DM complications. To our knowledge, no meta-analyses or reviews have investigated the efficacy and safety of metformin on cardiovascular outcomes after AMI in patients with T2DM. We thus conduct a high-quality systematic review and meta-analysis to assess the efficacy and safety of metformin on cardiovascular outcomes after AMI in patients with T2DM. It is hypothesized that metformin use at the post-AMI is associated with decreased risk of cardiovascular disease and death in patients with T2DM. The results of this research will be delivered in a peer-reviewed journal. This study expects to provide credible and scientific evidence for the efficacy and safety of metformin on cardiovascular outcomes after AMI in patients with T2DM.^[5]^

## Author contributions

**Conceptualization:** Jun Yang.

**Data curation:** Chuanwen Shen, Shuying Tan.

**Formal analysis:** Chuanwen Shen, Shuying Tan.

**Funding acquisition:** Jun Yang.

**Investigation:** Chuanwen Shen, Shuying Tan.

**Methodology:** Jun Yang.

**Resources:** Jun Yang.

**Software:** Chuanwen Shen, Shuying Tan.

**Supervision:** Jun Yang.

**Validation:** Chuanwen Shen, Shuying Tan.

**Visualization:** Shuying Tan.

**Writing – original draft:** Chuanwen Shen, Shuying Tan.

**Writing – review & editing:** Jun Yang.
